# Executive Functions and Child Psychopathology: Contextual Differences and Predictors for Detection and Psychoeducational Intervention

**DOI:** 10.3390/children12091217

**Published:** 2025-09-11

**Authors:** Juan Manuel Núñez, Ana Soto-Rubio, Marián Pérez-Marín

**Affiliations:** 1 Faculty of Psychology and Speech Therapy, Universitat de València, 46010 Valencia, Spain; nujuanma@alumni.uv.es; 2 Department of Developmental and Education Psychology, Universitat de València, 46010 Valencia, Spain; 3 Department of Personality, Assessment and Psychological Treatments, Universitat de València, 46010 Valencia, Spain; marian.perez@uv.es

**Keywords:** executive functions, child psychopathology, special educational needs, emotional and behavioural adjustment, school context, early detection, psychoeducational intervention, BRIEF-2, transdiagnostic predictors, primary education

## Abstract

Background/Objectives: Executive functions (EFs) play a fundamental role in children’s cognitive and emotional regulation and have been identified as key transdiagnostic predictors of psychopathology. Children with Special Educational Needs (SENs) are particularly vulnerable to difficulties with EFs and emotional–behavioural adjustment. This study aimed to examine the differences in the psychopathological symptoms between pupils with and without SENs and to explore the predictive ability of dimensions of EFs for psychopathology detection in both school and family contexts. Methods: A total of 123 primary school children (aged 6–12 years) participated in the study. Their psychopathology was assessed using the SPECI questionnaire completed by their teachers, while their EFs were measured using the BRIEF-2 from school and family perspectives. The analyses included mean difference tests and a backward stepwise multiple regression using the predictors that showed significant Pearson’s correlations with the psychopathological dimensions. Results: The students with SENs showed significantly higher levels of psychopathological symptoms, particularly in their attention, anxiety, and clinical global scores. The regression models revealed that several dimensions of EFs, such as inhibition, planning, task monitoring, and cognitive regulation, significantly predicted internalising, externalising, and total symptoms. The school-based models demonstrated greater explanatory power compared to the family-based models, suggesting that school contexts may be more sensitive for detecting EF-related difficulties. Conclusions: The results underline the transdiagnostic relevance of EFs in child psychopathology and support their integration into early detection and intervention strategies, especially in educational contexts. Strengthening the assessment of EFs in schools could contribute to a more accurate identification of at-risk pupils and inform targeted support for children with SENs.

## 1. Introduction

Executive function (EF) refers to a set of self-regulatory cognitive skills that include inhibitory control, working memory, cognitive flexibility, planning, and behavioural monitoring, among others [1]. These functions enable individuals to adapt to changing demands, regulate their emotions, achieve goals, and behave in socially appropriate ways, while also being directly related to people’s psychological well-being [2]. In recent decades, numerous studies have demonstrated a significant relationship between executive functioning and the onset, maintenance, and severity of various forms of psychopathology in childhood and adolescence [1].

From a developmental psychopathology perspective [3], it has been proposed that EF is central to the trajectory of adaptive development, as deficits in these skills may arise as a consequence of early adverse experiences and constitute a pathway toward the emergence of both internalising and externalising psychological problems [4,5]. Within this framework, and from a dimensional approach, a hierarchical bifactor model of psychopathology has been described, comprising a general factor—referred to as “p”—and lower-order specific factors, such as internalising problems (e.g., anxiety, depression) and externalising problems (e.g., disruptive behaviour, aggression) [6,7,8]. This general factor is estimated to explain more than 50% of the shared variance among symptoms and is considered a transdiagnostic vulnerability to multiple forms of psychopathology [1].

Longitudinal studies have shown the stability of the p-factor between the ages of 7 and 15 [9], as well as its evolution toward more specific disorders depending on the contextual and developmental interactions [10]. Among the most consistent predictors of a high score for this factor are difficulties in executive functions, particularly in tasks requiring emotional self-regulation, impulse control, and flexible adaptation to social norms [11,12]. From a neurodevelopmental perspective, a model has been proposed in which adverse childhood experiences disrupt the development of the neural systems that support EFs, thereby increasing the risk of general psychopathology. This model outlines a developmental sequence that includes the following: (a) exposure to stress and adversity, (b) the disruption of executive development, and (c) the emergence of transdiagnostic psychopathological symptoms [1,13].

EF deficits are associated with a wide variety of conditions that affect psychological well-being [2], including mental disorders, ADHD, autism spectrum disorder, affective disorders, anxiety, behavioural problems, and substance abuse [14]. A meta-analysis involving more than 16,000 individuals reported transdiagnostic reductions in the grey matter volume in regions such as the dorsal anterior cingulate cortex and bilateral anterior insula—areas implicated in EF, emotional regulation, and interoceptive integration—suggesting a common neural substrate underlying the clinically observed transdiagnostic vulnerability [1,15].

From an applied perspective, the relationship between EF and psychopathology is particularly relevant in educational contexts. In a study by Pagerols et al. (2022) [16], attention problems—closely linked to EF—significantly predicted lower academic achievement in a sample of more than 7000 children and adolescents. This association remained significant even after controlling for other psychopathological dimensions and contextual factors. Similarly, research by Visser et al. (2020) [17] found high comorbidity rates between specific learning disorders and ADHD, as well as with anxiety, depression, and conduct disorders. These comorbidities can be partially explained by EF impairments, which hinder both learning and emotional and behavioural self-regulation.

In the same vein, recent studies have explored how mental health problems negatively affect EF development, especially during childhood and adolescence. This is supported by the study of Frutos-de Miguel (2024) [18], who examined a sample of 689 students aged 10 to 14 years using a latent class analysis. The findings revealed that anxiety and depression were significantly associated with poorer performance in working memory, inhibitory control, and cognitive flexibility—all of which directly impact school performance and emotional self-regulation. These results highlight the need for educational interventions that address not only cognitive skills but also the emotional difficulties that interfere with them [19,20].

Likewise, the empirical evidence supports a bidirectional relationship between executive dysfunction and general psychopathology. Romer and Pizzagalli [21], in a longitudinal study of more than 9800 preadolescents from the Adolescent Brain Cognitive Development Study, found that lower EF scores predicted increases in the p-factor two years later, even when controlling for prior psychopathology. Conversely, elevated p-factor levels predicted subsequent impairments in EF. These results suggest that executive dysfunction may function both as a risk marker and as a consequence of general psychopathology. Furthermore, this association appears stable across different symptom domains, including externalising, internalising, neurodevelopmental, and somatisation problems. The stability of this link positions EF as a promising target for transdiagnostic preventive interventions [21].

Taken together, the current evidence reinforces the importance of executive functions as a key construct for understanding psychopathological development in childhood. From both clinical and educational perspectives, there is a clear need to implement preventive approaches that integrate the cognitive and emotional dimensions—especially in school settings, where such difficulties tend to manifest more clearly and systematically. Strengthening executive functioning thus emerges as a promising avenue for reducing the risk of mental disorders in childhood and adolescence, as well as for promoting more adaptive and resilient developmental trajectories.

Within this context, it is pertinent to further analyse the relationship between executive functions and psychopathology in school populations, considering the differences between the informant contexts (family vs. school) and the potential influence of Special Educational Needs (SENs).

Accordingly, the present study sets out the following objectives: (1) to analyse the differences in the levels of psychopathological symptoms between students with and without SENs, and (2) to examine the predictive value of different dimensions of EF on internalising, externalising, and overall psychopathological symptoms, taking into account the assessment context (family and school).

## 2. Materials and Methods

### 2.1. Procedure and Sample

The sample consisted of 123 primary school students aged 6 to 12 years, with the Mean (SD) = 8.61 (1.80) years, with a relatively homogeneous distribution across school years, averaging approximately 20 students per grade and ranging from 19 to 25. The participants were recruited from mainstream schools in the province of Castellón through agreements with the schools and the voluntary participation of families who provided informed consent.

Of the total, 53.6% were boys. According to the information provided by the teachers, 32% of the students had Special Educational Needs (SENs), a proportion higher than the 17.7% estimated in the Spanish primary school population [22], probably due to the greater involvement of families of children with these characteristics. Overall, 68% of the students did not present with SENs, whereas 32% did. Within this latter group, their needs were distributed as follows: learning difficulties, which accounted for 15.4% of the total sample (equivalent to 48.1% of students with SENs); attention difficulties, which were 4.9% of the total (15.4% of those with SENs); autism spectrum disorder (ASD), which was 3.3% of the total (10.3% of SENs); communication difficulties, which were 2.4% of the total (7.7% of SENs); intellectual disability, which was 0.8% of the total (2.6% of SENs); and 4.1% of the total (12.8% of SENs) was not specified.

The SENs variable was coded dichotomously (yes/no) due to its heterogeneity; nevertheless, all the students with SENs shared a similar schooling profile, as they attended mainstream schools, were in regular classrooms, and followed the general curriculum. In addition, 3.2% of participants had a medical diagnosis and 11.2% had a previous psychological diagnosis. In all cases, the students’ mother tongue included Spanish and/or Valencian, as the area is bilingual, and all students had the required level of Spanish to complete the tests.

Regarding specialised support within the school setting, 7.2% of the students received therapeutic pedagogical support and 8% received speech and language interventions. Concerning the support outside the school context, 60% did not receive any specialised interventions, while 3.2% received psychological support, 2.4% received pedagogical interventions, and another 2.4% received interdisciplinary interventions.

The study also included 88 parents, mostly mothers (82.9%), aged between 29 and 57 years, with the Mean (SD) = 41 (5.25) years. A total of 48% were married, and 33.6% had completed secondary education, while 24.8% had completed university studies. Regarding employment status, 28.8% had permanent contracts and 16.8% were self-employed.

Following institutional approval, a training session was conducted for the teachers and families in order to standardise the data collection procedure and ensure understanding of the instruments used. The school assessments were administered during a group tutoring session, while the family questionnaires were distributed and collected within a one-week period.

The study was approved by the Ethics Committee of the University of Valencia (reference UV-INV_ETICA-3119648) on 18 December 2023, and was conducted in accordance with the ethical principles of the Declaration of Helsinki (2013) and the provisions of the Spanish Organic Law on Personal Data Protection (LOPD 3/2018, of 5 December).

### 2.2. Instruments

Behaviour Rating Inventory of Executive Function–2 (BRIEF-2) [23]: A standardised instrument for assessing executive functions in children and adolescents aged 5 to 18 years, based on reports from parents and teachers. It consists of 63 items with three response options (never, sometimes, often), organised into nine clinical scales: inhibition, self-control, flexibility, emotional control, initiative, working memory, planning and organisation, task monitoring, and organisation of materials. These scales yield three composite indices (behavioural regulation index, emotional regulation index, and cognitive regulation index) and a Global Executive Composite. The instrument has been validated in the Spanish population and demonstrates adequate psychometric properties. In the present sample, the internal consistency was good: α = 0.97 for the family version and α = 0.98 for the school version.

SPECI (Screening of Emotional and Behavioural Problems in Children) [24]: A brief questionnaire designed to detect early indicators of child psychopathology in school populations aged 5 to 12 years, completed by teachers. It consists of 10 items assessing observable behaviours in the school context, categorised into seven specific dimensions: withdrawal, anxiety, dependence, dysfunctional thinking, attention problems, academic difficulties, and behavioural problems. These are grouped into three global scales: internalising problems (withdrawal, anxiety, and dependence), externalising problems (disruptive behaviour, attention problems), and a total psychopathology index (reflecting the overall level of emotional and behavioural difficulties observed in the school context). The instrument has been validated in the Spanish population and shows adequate psychometric indicators. In the present sample, the internal consistency for the SPECI was acceptable (α = 0.79).

Ad hoc register: Specifically developed for this study, this form was used to gather sociodemographic and clinical information about the children, their families, and their school tutors. The variables included age, sex, school year, medical and/or psychological diagnoses, parental education levels and employment status, and the type of specialised care the child receives both in and outside of school. SENs status was identified through the official curricular adaptation report issued by the school counsellors.

### 2.3. Variables

The independent variables were gender, age, and the presence or absence of Special Educational Needs (SENs), classified into two categories: “with SEN” and “without SEN.”

The dependent variables were grouped into two blocks:

Executive functions (assessed with the BRIEF-2): These included inhibition, self-control, flexibility, emotional control, initiative, working memory, planning and organisation, task monitoring, and organisation of materials. These were complemented by the three composite indices and the Global Executive Composite.

Child psychopathology (assessed with the SPECI): These included internalising problems, externalising problems, and total psychopathology, based on the teacher reports.

### 2.4. Design

This study employed a cross-sectional, comparative, and correlational design, aimed at analysing the associations between the executive function dimensions and psychopathological symptoms, as well as the differences according to Special Educational Needs (SENs) status.

## 3. Results

### 3.1. Difference in Means

Mean difference analyses were conducted to compare the levels of psychopathological symptomatology between the students with and without SENs. The results showed that no significant differences were observed for the scales of somatisation, conduct, depression, and violence. For the remaining dimensions, the differences were statistically significant, with higher scores in the SENs group (see Table 1). Only the scales with statistically significant differences are shown.

On the specific scales, the students with SENs were more affected by withdrawal, anxiety, dependence, atypical thinking, and attention and academic performance problems. The most significant differences between the groups were recorded for the dimensions of attention and anxiety.

As for the composite indices, significant differences were observed in the three domains—internalising problems, externalising problems, and the total psychopathology index—the latter being where the most significant difference between the two groups was observed.

### 3.2. Regressions

Multiple linear regression models were constructed using the backwards selection procedure, with the predictors limited to those variables that had demonstrated significant correlations with each dimension of psychopathology in the previous Pearson correlation analysis. This strategy allowed us to identify the most parsimonious set of predictor variables for each of the scales of the SPECI instrument, maximising the variance explained and minimising the residual error. The resulting regression models, chosen for each dimension of psychopathology in both contexts (family and school), were selected based on their explanatory power, overall statistical significance, and balance between complexity and fit. These models are described below (see Table 2).

The family context regression models demonstrated moderate explanatory power, with R^2^ values ranging from 0.295 to 0.342. The model with the best fit was that for internalising problems, followed by that for externalising problems and, finally, that for total problems. In all cases, the models were statistically significant, although the values of the F-statistic indicated moderate power.

In contrast, the models corresponding to the school context showed a significantly higher explanatory power. The internalising problems prediction model had an R^2^ of 0.753, with a high F-statistic indicating a high fit. The models for externalising and total problems also showed a good fit, with coefficients of determination of 0.507 and 0.647, respectively, and significant F-values. These results suggest that the predictor variables assessed in the school environment explain a greater proportion of the variance in psychopathology scores compared to the measures obtained in the family context.

In the family context, internalising problems were negatively associated with inhibition, planning, organisation, task monitoring, and organisation of materials, while they showed positive associations with flexibility, emotional control, and the cognitive regulation index. Regarding externalising problems, positive associations were observed with working memory, task monitoring, and the behavioural regulation index, and a negative association with the cognitive regulation index (see Table 3). Overall, the total problems showed negative associations with inhibition, initiative, planning and organisation, task monitoring, and organisation of materials.

In the school context, internalising problems showed negative associations with inhibition, emotional control, and the cognitive regulation index. In contrast, they were positively related to planning and organisation, the organisation of materials, and the behavioural regulation index (see Table 4).

Externalising problems were negatively associated with working memory, task monitoring, and organisation of materials, and positively associated with flexibility and the index of cognitive regulation.

In the case of total problems, the negative predictors were working memory and task monitoring, while positive associations were identified with the behavioural regulation index and the cognitive regulation index.

In both contexts, there was overlap in the relevance of functions, such as task monitoring, planning and organising, and organising materials, all of which were significantly related to different dimensions of psychopathology. The index of cognitive regulation was also identified as a predictor in all three models in both contexts, although its direction varied according to the dimension and setting.

Some differences were particularly striking: inhibition was significantly associated with internalising problems in the school context but not in the family context. Working memory was a significant predictor of externalising and total problems in the school context, while in the family context, it was only linked to externalising problems.

## 4. Discussion

The present study aimed to examine the relationship between executive functioning (EF) and psychopathological symptoms in school-aged children, with particular attention to the moderating or mediating role of Special Educational Needs (SENs). The results suggest that children with SENs tended to present with lower EF profiles and higher levels of internalising and externalising symptoms compared to their non-SENs peers, in line with previous research indicating the greater vulnerability of this population to cognitive and emotional dysregulation [21].

The regression models indicated that the EF variables were statistically significant predictors of both internalising and externalising symptoms, even after controlling for sociodemographic and contextual variables, such as parental education, family structure, and classroom environment. Nevertheless, these associations should be interpreted with caution. The EF information was obtained from parent and teacher reports using the BRIEF-2, which, while potentially subject to informant bias, also represents a strength in terms of ecological assessment by integrating two key contexts (family and school). This approach contrasts with traditional assessments based solely on student self-reports, offering a broader and more contextualised view of executive functioning in daily life.

The inclusion of SENs status in the models suggests a possible partial mediation effect, whereby children with SENs may be more sensitive to the emotional and behavioural consequences of EF difficulties. This pattern is consistent with developmental cascade models [2], which propose that early cognitive vulnerabilities interact with environmental stressors, increasing the risk of later psychopathological outcomes. Our findings extend previous research by showing that this association remains significant even in inclusive educational settings, suggesting that the mere presence of supportive classroom environments may not be sufficient to buffer the impact of EF difficulties in SENs students.

From an applied perspective, the results reinforce the relevance of early identification and targeted interventions aimed at strengthening EF skills, particularly in populations with SENs. School psychologists and educators should receive training to detect potential EF difficulties and to implement evidence-based supports, such as metacognitive training, scaffolding strategies, and behavioural self-regulation techniques. Furthermore, the adoption of a multi-tiered system of support (MTSS) integrating academic, behavioural, and emotional assessments could help deliver more individualised interventions [18].

Among the limitations, it should be noted that the cross-sectional design prevents causal inferences, and the absence of direct performance-based measures limits the interpretation of the findings in diagnostic terms. Nonetheless, the combined use of reports from two contexts and the comparative analysis of students with and without SENs add value, as they allow for the exploration of differential patterns of association depending on the observation setting—an approach rarely adopted in previous studies.

Despite these limitations, this work contributes to the evidence on the role of EF in child psychopathology and underscores the need for future longitudinal and multi-method research to examine the stability and directionality of these associations. Its value lies in having demonstrated this relationship within a doubly contextualised approach (family and school), but with the necessary interpretative reservations derived from the methodological limitations noted. This approach provides a perspective closer to the students’ everyday realities and facilitates the detection of difficulties that might go unnoticed in evaluations focused exclusively on a single context or on students’ self-reports.

In addition, the comparison between students with and without SENs in an inclusive framework provides relevant information for educational decision-making and for designing interventions more closely tailored to the real needs of each group. The study therefore offers clear practical implications for psychoeducational assessment and for the implementation of preventive programmes in school contexts, reinforcing the idea that EF assessment should be incorporated into early detection and educational planning protocols. This approach may contribute to optimising the available resources; prioritising students at greater risk; and improving coordination among families, teachers, and educational support professionals [1,18,21].

## 5. Conclusions

This study highlights the high prevalence of psychopathological symptomatology in students with SENs, as well as the relevance of executive functions as a transdiagnostic explanatory factor of emotional and behavioural problems in childhood. EFs are a significant predictor of internalising, externalising, and global symptoms, with the variables, such as task monitoring, planning, and the index of cognitive regulation, being particularly consistent.

The school context emerges as a key space for both identifying difficulties and implementing preventive and intervention strategies. The greater predictive capacity of school models underscores the need to strengthen the role of teachers in the psycho-pedagogical assessment process, especially in detecting alterations in cognitive and behavioural self-regulation.

These findings support the interest in incorporating measures of executive functioning into screening and initial assessment protocols to guide more effective and tailored educational interventions. They also invite the consideration of executive functions as a relevant construct in the development of transdiagnostic diagnostic tools.

Despite its limitations, the present study provides evidence of the predictive value of EF in childhood. It raises new lines of research focused on its role within longitudinal models, in which risk and protection trajectories can be analysed in relation to psychopathological development.

## Figures and Tables

**Table 1:** Difference in means in psychopathology (SPEC) between students with SENs and without SENs.

	NO SENs (*n* = 83) (Mean; SD)	YES SENs (*n* = 25) (Mean; SD)	*t*	*p*	*d*
Retraction	0.10; 0.297	0.36; 0.569	−2.228	0.034	0.376
Anxiety	0.06; 0.239	0.58; 0.717	−3.517	0.002	0.397
Dependent	0.01; 0.110	0.40; 0.500	−3.852	0.001	0.257
Thinking	0.00; 0.00	0.33; 0.702	−2.326	0.022	0.329
Attention	0.16; 0.398	0.58; 0.830	−2.440	0.022	0.524
Academic	0.02; 0.154	0.46; 0.658	−3.208	0.004	0.337
Internalising	0.27; 0.683	2.00; 1.902	−4.186	<0.001	1.050
Externalising	0.25; 0.730	1.52; 2.192	−2.734	0.012	1.119
Total	0.51; 0.980	3.59; 3.390	−4.222	<0.001	1.776

Notes: SD = standard deviation. Cohen’s d: small TE ≈ 0.20; moderate TE ≈ 0.50; large TE ≈ 0.80.

**Table 2:** Regression models: Family and school contexts as predictors of psychopathology.

	R^2^	R^2^ aj.	F	*p*
Models Family				
Internalising Psychopathology	0.342	0.253	3.817	<0.001
Externalising Psychopathology	0.322	0.228	3.426	0.002
Total Psychopathology	0.295	0.233	4.749	<0.001
Models School				
Internalising Psychopathology	0.753	0.734	39.969	<0.001
Externalising Psychopathology	0.507	0.470	13.541	<0.001
Total Psychopathology	0.647	0.628	34.123	<0.001

Note: R^2^ = coefficient of determination; adj. R^2^ = adjusted R^2^; F = F-statistic for the regression model.

**Table 3:** Predictors of family context in child psychopathology.

	B	SE	*t*	*p*
Model: Psychopathology Internalising Family				
Inhibition	−0.038	0.018	−2.137	0.036
Flexibility	0.233	0.134	2.447	0.017
Emotional control	0.307	0.185	2.839	0.006
Initiative	−0.035	0.026	−1.352	0.181
Planning and organisation	−0.088	0.032	−2.706	0.009
Task supervision	−0.062	0.023	−2.648	0.010
Organisation of materials	−0.068	0.021	−3.168	0.002
Emotional regulation index	−0.461	0.282	−1.634	0.107
Cognitive regulation index	0.225	0.058	2.610	<0.001
Model: Externalising Psychopathology Family				
Inhibition	−0.029	0.015	−1.971	0.053
Flexibility	−0.093	0.051	−1.838	0.071
Emotional control	−0.134	0.071	−1.893	0.063
Working memory	0.034	0.011	3.209	0.002
Planning and organisation	0.023	0.012	1.926	0.059
Task supervision	0.019	0.008	2.431	0.018
Behavioural regulation index	0.043	0.016	2.727	0.008
Emotional regulation index	0.191	0.107	1.784	0.079
Cognitive regulation index	−0.066	0.021	−3.140	0.003
Model: Total Psychopathology Family				
Inhibition	−0.045	0.018	−2.535	0.014
Initiative	−0.066	0.029	−2.278	0.026
Planning and organisation	−0.090	0.036	−2.493	0.015
Task supervision	−0.068	0.026	−2.600	0.011
Organisation of materials	−0.077	0.023	−3.298	0.002
Cognitive regulation index	0.280	0.065	4.344	<0.001

Note: B = unstandardised coefficient; SE = standard error; *t* = *t*-value of the model.

**Table 4:** Predictors of school context in child psychopathology.

	B	SE	*t*	*p*
Model: Psychopathology Internalising School				
Inhibition	−0.150	0.043	−3.487	<0.001
Self-monitoring	−0.051	0.026	−1.940	0.055
Emotional control	−0.031	0.011	−2.905	0.005
Planning and organisation	0.101	0.018	5.510	<0.001
Organisation of materials	0.034	0.010	3.264	0.002
Behavioural regulation index	0.236	0.063	3.773	<0.001
Cognitive regulation index	−0.052	0.018	−2.947	0.004
Model: Psychopathology School				
Flexibility	0.065	0.031	2.077	0.041
Working memory	−0.110	0.053	−2.081	0.040
Planning and organisation	−0.065	0.039	−1.679	0.097
Task supervision	−0.086	0.031	−2.831	0.006
Organisation of materials	−0.054	0.019	−2.856	0.005
Emotional regulation index	−0.024	0.030	−0.790	0.432
Cognitive regulation index	0.321	0.105	3.055	0.003
Model: Psychopathology Totals School				
Flexibility	0.034	0.017	1.947	0.055
Working memory	−0.153	0.057	−2.700	0.008
Task supervision	−0.102	0.032	−3.166	0.002
Behavioural regulation index	0.027	0.011	2.433	0.017
Cognitive regulation index	0.362	0.081	4.497	<0.001

Note: B = unstandardised coefficient; SE = standard error; *t* = *t*-value of the model.

## Data Availability

The data presented in this study are available on request from the corresponding author. They are not publicly available due to data privacy and confidentiality.
